# Smoothness: an Unexplored Window into Coordinated Running Proficiency

**DOI:** 10.1186/s40798-019-0215-y

**Published:** 2019-11-09

**Authors:** John Kiely, Craig Pickering, David J. Collins

**Affiliations:** 1 Institute of Coaching and Performance, School of Sport and Health Sciences, University of Central Lancashire, Preston, UK; 2 Athletics Australia, Brisbane, Queensland Australia; 3 Grey Matters Performance Ltd., Birmingham, UK; 4 Moray House School of Education and Sport, University of Edinburgh, Edinburgh, UK

## Abstract

Over the expanse of evolutionary history, humans, and predecessor *Homo* species, ran to survive. This legacy is reflected in many deeply and irrevocably embedded neurological and biological design features, features which shape how we run, yet were themselves shaped by running.

Smoothness is a widely recognised feature of healthy, proficient movement. Nevertheless, although the term ‘smoothness’ is commonly used to describe skilled athletic movement within practical sporting contexts, it is rarely specifically defined, is rarely quantified and remains barely explored experimentally. Elsewhere, however, within various health-related and neuro-physiological domains, many manifestations of movement smoothness have been extensively investigated. Within this literature, smoothness is considered a reflection of a healthy central nervous system (CNS) and is implicitly associated with practiced coordinated proficiency; ‘non-smooth’ movement, in contrast, is considered a consequence of pathological, un-practiced or otherwise inhibited motor control.

Despite the ubiquity of running across human cultures, however, and the apparent importance of smoothness as a fundamental feature of healthy movement control, to date, no theoretical framework linking the phenomenon of movement smoothness to running proficiency has been proposed. Such a framework could, however, provide a novel lens through which to contextualise the deep underlying nature of coordinated running control. Here, we consider the relevant evidence and suggest how running smoothness may integrate with other related concepts such as complexity, entropy and variability. Finally, we suggest that these insights may provide new means of coherently conceptualising running coordination, may guide future research directions, and may productively inform practical coaching philosophies.

## Key Points

- Smoothness is a universal feature of healthy skilled movement which, although infrequently considered and currently under-appreciated within sporting contexts, may provide a unique window into athletic coordinative proficiency.
- Existing evidence illustrates that smoothness changes as a consequence of natural aging, general health status, practice and injury history and current fatigue and injury status. Preliminary research suggests that running proficiency is reflected in smoother running movement.
- Recent advances in wearable technologies provide the opportunity to sensitively detect changes in running smoothness, thereby potentially bestowing unique insights into running coordination proficiency

## Introduction: What Do Running Proficiency and Hard-Core Pornography Have in Common?

During his tenure as a US Supreme Court justice, Potter Stewart presided over many high profile cases. He, for example, promoted personal privacy protections and extended the 1866 Civil Rights Act to concede that schools should not discriminate on the basis of race. Outside of legal contexts, however, he is best remembered for a single clause, from a single sentence. While adjudicating on the legality of the state of Ohio’s banning of an allegedly pornographic film, Stewart uttered perhaps the most famous phrase in the Supreme Court history, ‘I shall not today attempt further to define the kinds of material I understand to be embraced within that shorthand description [“hard-core pornography”], and perhaps I could never succeed in intelligibly doing so. But I know it when I see it … [1]’ In this context, ‘I know it when I see it’ is a euphemistic abstraction describing a phenomenon that—by virtue of its apparent ‘obviousness’—is simultaneously familiar to all, yet surprisingly difficult to characterise, quantify or elegantly articulate.

The topic explored in this article, we suggest, shares these features in that although, superficially, it appears intuitively obvious and readily apparent; when we attempt to explain exactly what ‘it’ is, we find that beneath this facade of familiarity lies a phenomenon that remains inadequately defined and poorly understood.

### What Is Movement Smoothness?

Across a diversity of literatures, and within practical coaching contexts, movement smoothness is generally recognised as a universal feature of skilled motor behaviour [2]. Yet, despite this assumed association between movement smoothness and movement proficiency, current definitions of smoothness remain surprisingly vague. A recently proposed definition suggests that a movement is perceived to be smooth when it happens in a continual fashion without any interruptions, suggesting smoothness is a quality reflecting the continuality or non-intermittency of movements that alternately accelerate and decelerate, and thereby remains independent of amplitude and duration [3]. In this context, more intermittency corresponds to less smoothness, and less intermittency to smoother movement.

The minimum-jerk model, first proposed by Flash and Hogan, suggested that human movement is executed in a manner that optimises smoothness by minimising its opposite, kinematic jerk [4]. Although smoothness can be assessed in multiple ways—a recent review suggests at least eight methods have been used in research contexts—the most common means of assessing smoothness is through the quantification of jerk [3]. Jerk is formally defined as the rate of change in acceleration, in mathematical terms, the first time derivative of acceleration, the second time derivative of velocity and the third time derivative of position [4, 5]. The smoothest movements consequently have, by definition, the lowest jerk [6]. Accordingly, within the neuroscientific literature, smooth movement has been described as any movement that is not ‘jerky’ [7].

From a practical coaching perspective, however, we can sensibly broaden this definition by proposing that smooth movements are those without abrupt, intermittent, discontinuous changes in accelerations, relative joint positions and/or movement trajectories. Accordingly, although movements may occur rapidly, unexpectedly, even violently, there is a sense of consistent flow, of finely regulated progression and seamlessly continuous coordinative control. Thus, key dimensions of movement—postural control, relative joint positions, the absorption of impacts—all appear to rhythmically and incrementally rise and fall. Visually, accordingly, we register a sense of fluency as the athlete dynamically progresses through a given movement sequence. Non-smooth movements, in contrast, leave an impression of abruptness, of erratic discordance and of disjointed, unpredictable control.

Smoothness seems intuitively recognised as a hallmark of skilled, coordinated movement [8]. Nevertheless, in relation to sporting movements in general, and running specifically, although the term ‘smoothness’ is commonly used to describe performer’s movement ability, it is rarely defined, is rarely empirically quantified, is barely explored academically and is typically not directly targeted in training. In short, running smoothness is a phenomenon that we instinctively ‘feel’ we recognise when watching elite performance. Yet, beyond this intuitive recognition, exactly what smoothness is remains surprisingly vague. Thus, just as Potter Stewart struggled to eloquently articulate the essence of a phenomenon as superficially self-evident as pornography, we similarly struggle to accurately characterise a dimension of movement as seemingly obvious as smoothness.

Notably, recent advances in accelerometer technology now provide access to raw, unfiltered acceleration time series that can readily be converted to jerk data. Surprisingly, however, this research topic has received very little attention within sports science contexts. In attempting to enhance our appreciation of this potentially important, yet largely ignored phenomenon, here, we examine the general evidence relating to movement smoothness, before subsequently reflecting on how these insights may contribute to a more robust understanding of running coordination.

## The Progression and Regression of Movement Smoothness

Smoothness increases progressively as we transition from infant, to developing child, to mature adult, and regresses as we move from adult maturity into old age [9–11]. Furthermore, smoothness—whether assessed in gross movements or fine motor skills—improves, in logarithmic fashion, in parallel with the number of practice trials performed [12, 13]. This effect is such that practice-driven improvements are reflected in increased smoothness in movement tasks as diverse as walking [13], writing [14], rock climbing [15], driving a golf ball [16], piano playing [17], wheelchair propulsion [18], dancing [19], over-arm throwing [20] and in the hand dexterity of surgeons [21, 22].

Many dimensions of declining function are, conversely, reflected in the deterioration of movement smoothness. Most obviously, smoothness is compromised following neurological damage, such as stroke, and subsequent recovery is typified by the gradual restoration of smoother movement [13]. This effect is such that even simple measures of smoothness—evaluated in sit-to-stand tests, for example—can distinguish between older adults at risk of falls, older adults who are not a falls risk and younger adults [23, 24]. Similarly, smoothness during lifting movements—assessed at hip and ankle—declines with advancing age [25], and many disease states, such as Parkinson’s and Huntington’s, are accompanied by deteriorating smoothness [26]. Furthermore, in children, developmental disorders such as autism and Asperger’s are typified by a lack of movement smoothness [27], and smoothness measures accurately detect delayed motor skill acquisition [8].

Additionally, smoothness measures can discern between those who have previously suffered cervical injury and non-previously injured controls [28], between those feigning whiplash injury and sincere patients [29] and between wheelchair users with, or without, shoulder pain [18]. Smoothness assessments are also sufficiently sensitive to detect decrements in highly learned skills caused by, for example, the influence of distractions on the driving performance of experienced taxi drivers [30] and movement skill inhibition following emotional disturbances [31].

### Why Is Smoothness a Universal Feature of Human Movement?

Several theories of motor control hypothesise that the brain coordinates muscle activation patterns to minimise a single, task-relevant *cost function*. Historically, it was assumed that the most heavily prioritised cost function, shaping movement control, was energetic expenditure [32]. Recent investigations, however, clearly demonstrate that although energy conservation is unquestionably a consideration, it is neither the only, nor necessarily the dominant, cost function shaping motor behaviours [32, 33]. Modelling predictions, for example, illustrate that ‘impulsive running’—running with infinitely stiff, straight legs and zero sweep angle—minimises the mechanical cost of transport [34]. Nevertheless, we run with energetically costly, compliant legs in a manner deviating substantially from this hypothetical optimum [35]. In fact, energy conservation appears to be only one of a growing list of proposed constraints, each capable of adequately predicting the common kinematics of human movement. Such considerations include, for example, preservation of stability, reductions in the neural ‘effort’ expended in controlling movement, the minimisation of changes in torque, the minimisation of discomfort and the regulation of movement accuracy [3, 36, 37]. Thus, although practiced movements are typically executed in a manner that reduces energy costs, energy expenditure is not an exclusively over-riding priority and is certainly not minimised. Although, experimentally, it seems impossible to determine which cost function is most heavily prioritised by the brain, notably, models prioritising smoothness consistently produce high-performing predictions [3, 33, 34].

Nevertheless, although various rationales have been proposed within the relevant literatures, the reasons why smoothness is such a fundamental feature of healthy movement remain unclear. Previous research suggested that smoothness, during ground contact events, is an indirect consequence of the CNS’s preference to employ single activation signalling bursts to individual muscles [38]. The authors speculated that this strategy enabled adequate outcomes, while greatly simplifying neural control complexity. Furthermore, the authors noted they could see no reason why smooth movements offered advantages over non-smooth ones. Their proposal, instead, was that smoothness evolves naturally from the interplay between a single-stimulation-burst-per-muscle activation pattern, the linear behaviour of the leg spring and the innate viscoelastic and geometric properties of the musculoskeletal system. In essence, suggesting smoothness, in landing tasks, emerges as a by-product of an evolutionary preference for simplified neural control, rather than because smoothness, in and of itself, offers any additional benefits [38].

More recent work, however, has proposed that smooth movements are inherently more predictable than less smooth, more erratic ones [39]. A more accurate prediction of upcoming movement demands is of substantial benefit as it permits a more fine-grained alignment between forecasted demands, advance preparation to meet these demands and actually imposed demands [39]. Enhanced predictive accuracy, accordingly, facilitates a more precisely attuned—more timely and more finely calibrated—preparation for impending challenge. Accordingly, it is suggested that smoothness, as it promotes predictability, minimises movement error [39–41]. Similarly, more sensitive detection of subtle deviations from predicted trajectories facilitates more sensitive remedial adjustments, thereby offsetting the need for periodic, larger, more disruptive and energetically demanding corrective interventions [42].

Non-smooth (by definition, more jerky) movements, in contrast, are inherently less predictable [41]. This diminished predictability inevitably detracts from the accurate forecasting of the likely kinetic and kinematic consequences of upcoming ground contacts [43]. Any loss of calibration between anticipated and actually imposed demands inevitably leads to larger deviations from expected trajectories, thereby requiring more drastic remedial interventions to ‘correct’ unwanted deviations [39]. Larger corrective interventions necessitate larger motor commands, which generate, as a natural by-product, more signal-dependent neural noise, thereby further diminishing movement proficiency [43]. Consequently, previous evidence has been interpreted as suggesting that humans strive to optimise smoothness and minimise jerk [41].

In summary, more precise predictability facilitates a finer calibration between current preparation for soon-to-be-imposed demands and the likely extent of those challenges. Smooth movements, as they require smaller on-line course corrections, minimise the disruptive effects of signal-dependent noise emerging as a natural consequence of larger motor commands [39]. Consequently, in a mutually re-enforcing manner, smoothness enhances prediction and prediction enhances smoothness. Smoothness, accordingly, by facilitating improved prediction, minimises the necessity of persistent remedial correction and thus serves to, simultaneously, reduce both the neuronal computational burden associated with complex movement and energetic expenditure [3, 39, 40].

### The Foundations of Movement Smoothness

Locomotion is initiated by commands originating in the motor cortex [42]. These descending commands are mediated and modulated by control centres in mid-brain and brain stem, before subsequently activating spinally located central pattern generating (CPG) networks responsible for controlling the rhythmic synchronisation of the arms and legs, thereby delegating much of the coordination burden to lower, less evolutionarily expensive, neural control centres [42]. As rhythmic locomotion progresses, streams of sensory feedback return to spinal centres and serve to (a) guide the on-going customization of CPG outputs to current contexts and (b) trigger stabilisation reflexes [42]. Through these mechanisms, sensory feedback directly alters on-going feedforward activation, and changes in activation inescapably alter changes in sensation. These feedback and feedforward loops are so inseparably entwined that representing them as isolated entities seems no longer sensible. Instead, feedback and feedforward information flows are best perceived as wholly integrated, mutually modulating arms of the sensorimotor system [36, 37].

Inevitably, however, neural and reflex-activating feedback and feedforward loops take time and cannot instantaneously respond to imposed perturbation. Proficient execution of impact-dependent movements—walking, running, jumping—thus requires that the earliest remedial compensations, upon ground contact, are mediated by the practiced manipulation of the intrinsic material and structural properties of biological tissue collectives [41, 42, 44]. When skillfully deployed, the innate viscoelastic and geometrical properties of the running leg provide an instantaneous, non-neurological, yet skilled, response to impact perturbations (for little energetic and neurological investment) [41, 42]. Thus, informed by anticipation and actioned by feedforward instruction, the time-lag deficits implicit in top-down neurally mediated motor control are offset by the skilled manipulation of biological tissue properties [42, 45]. Rhythmic locomotion, accordingly, is regulated by the blended output of three distinct, but mutually and irrevocably entangled, levels of control:

1. Top-down, supra-spinal executive direction
2. Spinally located CPGs and stabilisation reflexes
3. The bottom-up, self-stabilising capacities afforded by the innate perturbation-resilient characteristics of bio-composite tissue structures [41]

When operating effectively, feedback and feedforward information is blended with the plastically embedded legacy of prior experience, to facilitate the skilled deployment of robust, task-conditioned, bio-composite tissue capacities. The fusion of these multi-level control systems underpins the runner’s ability to sensitively detect and respond to upcoming perturbations in ways that minimally disrupt rhythmical locomotion. Smoothness thus emerges as a natural outcome of this intimate integration between accurate anticipation of upcoming perturbations and the advance remediation of forecasted de-stabilisations [46, 47].

## Is Movement Smoothness an Important Feature of Running?

Within a number of academic literatures, smoothness is acknowledged as a fundamental characteristic of goal-directed human movement [3, 48]. Although not well investigated within sporting contexts, preliminary evidence suggests smoothness measures are capable of discerning between different levels of expertise. The clubhead trajectories of skilled golfers, for example, are smoother than those of unskilled golfers [49]. Recent research, furthermore, established that a lack of smoothness—in the postural sway adjustments of NCAA Division 1 College football players—predicted the likelihood of subsequent injury [50]. Such findings suggest smoothness is a phenomenon reflecting both practice-related skill improvements and the underpinning functional health of the neuro-muscular system.

Specifically, in relation to running, however, empirical insights remain sparse. An early study, by Hreljac, used video analysis techniques to determine runner’s jerk-cost at ground contact and established that competitive runners ran more smoothly than recreational runners [12]. Subsequently, Cortes and colleagues, using trunk-mounted sensors to collect acceleration data during a running-and-cutting maneuver, illustrated that fatigue-induced changes in motor variability detracted from the smooth execution of the target movement [51].

### Running Smoothness and the Loss of Complexity Hypothesis

In 1992, Lipsitz and Goldberger published an influential, and much cited, *JAMA* paper proposing the *loss of complexity hypothesis*, suggesting that, as we age, the complexly intertwined neural and biological foundations, which support all essential neurophysiological processes, gradually and progressively degrade [52]. Through this conceptual lens, reductions in complexity are indicative of declining neurophysiological responsiveness and adaptive range [52–54]. Interestingly, a limited number of recent investigations, using measures of *entropy*—a means of analysing the complexity inherent in a data time series—have demonstrated that running-induced fatigue changes the complexity of the acceleration signals emanating from sensors attached to a site approximating centre of mass (CoM) [55–57].

Translating the loss of complexity hypothesis to running contexts suggests that reductions in underlying neurobiological complexity diminish the spectrum of viable movement permutations capable of equitably providing equivalent stride outcomes for a comparable ‘cost’. Accordingly, changes in signal complexity are interpreted as reflecting a contracting range of available micro-movement permutations capable of collaboratively solving the running-imposed challenge [54]. Consequently, as complexity contracts, the inter-stride variability inherent in each runner’s stride pattern is impelled to dysfunctionally diverge from habituated norms [54, 58, 59]. This divergence, in turn, is hypothesised to expose the runner to both declining movement efficiency and exacerbated risk [51, 58, 59].

The relevance of this rationalisation, to the topic of running smoothness, is to suggest that diminishing neurobiological complexity—induced, for example, by fatigue, prior injury, pain sensitization and/or age-related decline—drives deteriorating coordinative control and the subsequent erosion of running smoothness [54, 58, 59]. As encapsulated within the loss of complexity hypothesis, the inevitable accumulation of experience-dependent wear and tear—associated with natural aging, declining health and injury and illness—progressively erodes both tissue micro-architectures and the connective integrity of densely entangled neural communications networks. Any subsequent reduction in neural communicative clarity—driven, for example, by injury, sensitization and/or residual fatigue—inevitably dims the runner’s fine-grained perception of their precise kinematic and kinetic context. (Although research in this realm remains sparse, prior injury has been observed to erode the proprioceptive capacities of elite runners and ballet dancers [60, 61]).

As a direct consequence, this diminished sensorimotor capacity impedes optimal preparation for upcoming ground contact and detracts from the sensitive calibration of running stiffness to the precise demands of the impact challenge [54, 59]. Consequently, reduced sensorimotor sensitivity directly diminishes coordinated control and can be expected to increase the magnitude of unexpected deviations from projected trajectories, thereby suggesting that diminished proprioception directly impedes activation precision and degrades movement smoothness [62–64]. Specifically, in running contexts, it has recently been suggested that the gradual degradation of available complexity is compounded by cycles of overuse, underuse, misuse and disuse [54, 65]. As complexity contracts, running coordination inevitably declines, and running smoothness deteriorates. Although the exact mechanisms underpinning this progressive deterioration remain unclear, two broad inter-related neuro-motor deficits have been implicated:

i. As the plastically embedded legacies of past cycles of injury, misuse, disuse and overuse accumulate within the CNS, the micro-structures underpinning neuronal connectivity progressively degrade, and available complexity contracts [54, 58, 59]. Consequently, sensorimotor communication clarity erodes, and both the interpretation of sensory feedback and the precision of feedforward activations gradually decay.
ii. Declining muscular strength—driven by neural signalling decrements, decreasing muscle mass and the degradation of tissue micro-structures—necessitates that, to adequately execute a task requiring a given movement force, weaker muscles require more relative activation, and hence larger activation signals than stronger muscles [54, 66]. Inevitably, larger relative activations result in increasing neural noise, thereby resulting in more disorderly motor unit recruitment and more erratically variable force outputs.

As multiple aspects of sensorimotor control—sensory acuity, activation accuracy and the load management capacity of biological tissues—erode, subsequent to the accumulating legacy of past insults, underlying complexity inevitably deteriorates. Accordingly, the spectrum of available coordinative responses to running-imposed mechanical challenges declines [67, 68]; consequently, smoothness declines. Although this proposed causal chain—linking neurobiological complexity, entropy, variability and running smoothness—appears theoretically robust, and has been observed in other movement contexts, it remains barely explored and has not been validated within human running applications [69].

## Future (Practical and Research) Directions?

Conceptually, smoothness seems an important facet of proficient running and a potential sensitive indicator of injury or fatigue-induced running deterioration. Clearly, however, evidence is lacking and the topic remains under-explored. We can, however, draw some speculative, but logical, initial conclusions:

1. Smoothness is modified by a range of factors, including:
  - Underlying health status (including neuro-physiological, psycho-emotional and disease status)
  - Training and injury history
  - Current fatigue and/or psycho-emotional states
2. Smoothness progresses and regresses as a function of normal maturation and aging, injury and subsequent recovery, and declining smoothness exposes tissues to exacerbated mechanical stress
3. Smoothness is a product of proficient coordination, mediated by the CNS, and actioned via the skilled deployment of the innate perturbation-resilient capacities of robust biological tissues

Given the recent evolution and current ubiquity of lightweight and sensitive accelerometer sensor technology, the evidence and rationalisations provided here also point towards potentially fruitful future research directions, highlighting, for example:

1. The opportunity to more fully explore the associations between fatigue, prior injury and running smoothness
2. The prospect of analysing acceleration time series using entropy-based techniques to better illuminate the hypothesised relationships between complexity, inter-stride variability, running proficiency and prior injury profiles
3. The proposition that enhanced accelerometer technology, entropy analysis techniques and the current availability of extensive computational capacity all suggest that we may be on the threshold of a transformation in how we conventionally devise, prescribe and monitor fatigue within running training contexts

Specifically, in relation to targeting running smoothness within training design and prescription contexts, beyond the general observation that running practice—under healthy, non-fatigued conditions—appears to enhance smoothness, there are no specific evidence-led guidelines. Speculation based on the sparse existing evidence does, however, hint that while practice improves, excessively repetitive practice leads to deteriorating neural communications and declining movement smoothness [42, 54]. Accordingly, more volume is not necessarily better. Instead, as with other facets of training management, improving running smoothness likely requires the sensitive regulation of volumes, intensities, exercise variation and the judicious balancing of work and recovery.

From a conditioning perspective, again, evidence is lacking and once more we are limited to theory-based speculation. Building on the rationale presented here, and elsewhere [54], we suggest smoothness is promoted by three broad categories of training intervention:

1. Engaging in the simple act of running at a range of paces and/or at target race pace under healthy, non-fatigued and non-sensitised conditions
2. Engaging in running-related challenges promoting an enhanced calibration between feedforward activation and proprioceptive information by providing non-habituated, coordination challenges capable of stimulating neuro-plastic re-modelling processes serving to refine communicative clarity between CNS and the peripheral musculature [54, 70, 71]
3. Engaging in training strategies serving to upgrade the structural and material resilience of biological tissues habitually subjected to mechanical stress during running activities [41], for example, resistance loading strategies [72], and/or strategies promoting more finely calibrated joint control, for example, dynamic stability challenges [73–75]

## Conclusion

Smoothness is a product of the collaborative triangulation between accurately interpreted sensory feedback and sensitively adjusted feedforward activation, contextualised against plastically embedded prior learning. As physical capacities and movement experiences accumulate, we innately gravitate towards smoother movement solutions as we learn to more sensitively respond to small perturbations, thereby offsetting the need to periodically and ‘jerkily’ respond to the larger challenges that would emerge if minor errors were allowed to accumulate. Smoothness thus reflects sensorimotor coordination and provides a quantifiable window into movement proficiency [5].

The rapid evolution of wearable micro-technology provides us with opportunities to accurately, and non-invasively, evaluate running smoothness. Currently, however, although evidence strongly suggests smoothness metrics provide insights into coordination proficiency, and can be used as markers of neuro-rehabilitation effectiveness, the most appropriate means to measure, monitor and analyse smoothness remain unclear [3, 48].

And so, just as Potter Stewart struggled to eloquently articulate the essence of a phenomenon as superficially self-evident as pornography, within both practical and theoretical running contexts, we similarly struggle to define and describe a phenomenon as intuitively familiar, yet as seemingly important as running smoothness. Although preliminary evidence demonstrates the informational value of smoothness assessments, such measures exist only on the periphery of our sporting cultural consciousness and remain poorly articulated, poorly understood and poorly explored.

Pornography may forever remain subjectively ambiguous and objectively unquantifiable, but that need not be the case with running smoothness. Yet, as discussed, an evidence-led logic supports the potential worth of objective smoothness evaluations, and currently, there is ready access to technologies enabling such evaluations. And while, unquestionably, much remains to be clarified and further research is (as always) necessary, the background and rationale outlined here serves as a useful conceptual starting point from where to begin this exploration.

## Data Availability

Not applicable.
